# Case report: A rare combination of right aortic arch with right patent ductus arteriosus and right tracheal bronchus causing impaired respiratory function

**DOI:** 10.3389/fcvm.2022.915111

**Published:** 2022-08-05

**Authors:** Xingchen Lian, Ning Wang, Chuan Bai, Ping Wen, Yuhang Liu

**Affiliations:** ^1^Department of Cardiothoracic Surgery, Dalian Municipal Women and Children’s Medical Center (Group), Dalian, China; ^2^Graduate School, Dalian Medical University, Dalian, China

**Keywords:** right aortic arch, right patent ductus arteriosus, right tracheal bronchus, case report, tracheobronchial stenosis

## Abstract

A right aortic arch with concomitant right patent ductus arteriosus and right tracheal bronchus is a rare congenital anomaly. Herein, the respiratory and circulatory functions of the child were normal at early ages, and imaging examination indicated that conservative treatment was suitable. However, with the growth and development of the child, the right tracheal bronchus was oppressed by the right arterial duct. We performed a cut and ligation of the right patent ductus arteriosus to relieve the pressure on the right tracheal bronchus. At the 6-month follow-up, the child had recovered well and exhibited no symptoms of respiratory restriction. Therefore, we believe that early interventions should be considered for this rare anatomic presentation to benefit the patient’s respiratory and circulatory systems. Our experience provides a foundational reference for future cases.

## Introduction

A right aortic arch with a left patent ductus arteriosus is likely to generate a clinically significant vascular ring that narrows the airway. However, a right aortic arch with a right patent arterial duct, as part of a normal anatomic variation, is associated with a relatively low risk of extracardiac anomalies or surgery (1). In this case, compression of the right tracheal bronchus by the right aortic arch with the right patent ductus arteriosus was identified by computed tomography angiography (CTA). We performed surgical ligation of the right patent ductus arteriosus to relieve airway compression in the patient. At the 6-month follow-up, no aberrant clinical symptoms were noted.

## Case description

During the prenatal phase, echocardiographs revealed that the fetus had a right aortic arch with a right patent ductus arteriosus (≤1 mm). Hepatitis B, human immunodeficiency virus, syphilis, and rubella tests were all negative in maternal prenatal tests. After birth, the infant performed well, with Apgar scores of 8 and 9 at 1 and 5 min, respectively. Transthoracic echocardiography and CTA were conducted after delivery to validate the findings of prenatal echocardiography and confirmed the right aortic arch with the right patent ductus arteriosus and the right tracheal bronchus. No interventions were carried out, considering that the infant did not exhibit any respiratory symptoms; however, follow-up after delivery was proposed. At the subsequent follow-up, transthoracic echocardiography showed that the right patent ductus arteriosus (≤1 mm) persisted. Family members were disinclined to surgical intervention, so further follow-up and observation were recommended.

**FIGURE 1:**
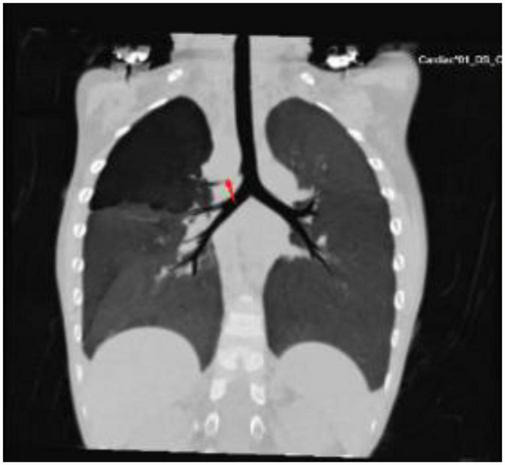
Computed tomography of the chest showed that the right upper lung had enhanced transmittance due to the right tracheal bronchus’ oppression (arrow).

**FIGURE 2:**
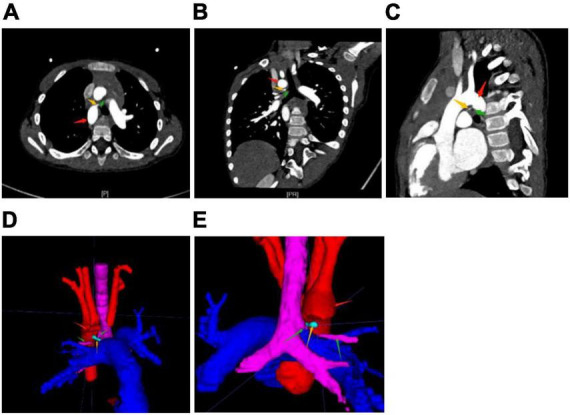
Computed tomography angiography [CTA, **(A–C)** horizontal, coronal, and sagittal] and [3-D reconstruction **(D,E)** front, back] show the right aortic arch (red arrow) and right ductus arteriosus (yellow arrow) with right tracheal bronchus (green arrow). The aorta and pulmonary artery are located in an anteroposterior relationship. In a normal conformation, even with the right aortic arch, the aorta is located to the right of the pulmonary artery. In addition, the left brachiocephalic artery and the right common carotid artery have a common trunk. This is the so-called bovine arch, a normal variant. The right tracheal bronchus, from the tracheal wall above the carina, runs in the “H”-shaped structure formed by the right aortic arch, right patent ductus, and right pulmonary artery.

Years later, the parents brought their 8-year-old child to our hospital under the mistaken assumption of pneumonia. Physical examination revealed hyperresonance in the right upper lung, and chest CT showed that the right upper lung had enhanced transmittance (Figure 1). Echocardiographs showed that the intra-cardiac anatomy was otherwise normal, in addition to good cardiac function, normal pulmonary artery, and right aortic arch with right patent ductus arteriosus (≤1 mm). On CTA, we found that the right aortic arch, right patent ductus arteriosus, and right pulmonary artery formed an “H-shaped” loop in conjunction with the right tracheal bronchus, which was “clamped” in it (Figure 2).

After informing the parents of the patient’s condition and related risks in detail, they requested urgent surgical intervention. Cut and ligation of the right patent ductus arteriosus through midline sternotomy without cardiopulmonary bypass was performed. At the 6-month follow-up, the patient had recovered well without any respiratory or cardiovascular symptoms. Table 1 summarizes the clinical presentation and management of the patient.

**TABLE 1:** Timeline of events.

Timeline	Events
2011	A male infant was born who had a right aortic arch with a right patent ductus arteriosus (≤1 mm).
2011∼2021.09	The child was regularly followed up in the hospital and no abnormal manifestations were observed.
2021.09	Imaging revealed the child’s airway obstruction and surgery was performed.
2021.09∼2022.03	On a follow-up of 6 months, the child recovered well without any symptoms of respiratory or cardiovascular problems.

## Discussion

The right aortic arch can be defined as an anatomic anomaly, wherein the aorta arches over the right side of the bronchus instead of the left. The right aortic arch grows from the right fourth pharyngeal arch artery and the right dorsal aorta embryologically. The prevalence of the right aortic arch is approximately 0.1% in the general population and 13–34% in the tetralogy of Fallot (2, 3). Bronchus suis, also known as pig bronchus or tracheal bronchus, is usually an asymptomatic abnormal bronchus that arises from the tracheal wall above the carina. The incidence of bronchus suis in humans is 0.1–3.0% (4). During early embryogenesis, an aberrant bronchus is normally the result of additional tracheal outgrowth (5).

**FIGURE 3:**
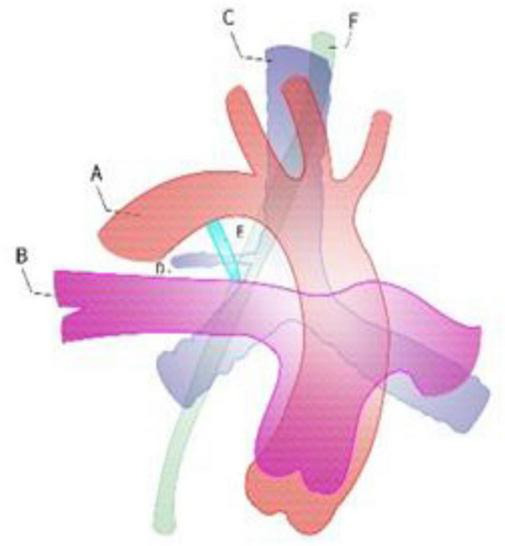
Right aortic arch with the right ductus against the right tracheal bronchus. A: descending aorta, B: right pulmonary artery, C: trachea, D: tracheal bronchus, E: right patent ductus, F: esophageal.

Right-sided ductus arteriosus occurs in 10% of fetuses with a right aortic arch and is usually asymptomatic (6). However, to the best of our knowledge, the combination of the right tracheal bronchus compressed by the right aortic arch with the right patent ductus arteriosus was reported herein for the first time. In our case, the right tracheal bronchus runs in an “H-shaped” conformation formed by the right aortic arch, right patent ductus, and right pulmonary artery (Figure 3). These anatomic variants are usually not clinically significant when they are present alone.

As the patient grew and developed, the tracheal bronchus became compressed and narrowed. At the early follow-up, we were inclined toward conservative treatment as the patient did not exhibit any symptoms. In addition, the follow-up observation was recommended in accordance with the families’ desire for conservative treatment. After the patient developed symptoms of airway obstruction, the family requested urgent treatment resolution. Post-case evaluation, the recommended treatment, included dissecting the ductus arteriosus or the arterial ligament and dissociating the surrounding tracheobronchial tissue to release the compression of the tracheal bronchus. The patient exhibited good postoperative recovery that confirmed our treatment strategy. However, further research is needed to determine whether earlier surgical intervention is warranted. In general, we should pay greater attention to the existence of airway blockages in patients when confronted with this unique anatomic combination.

## Data availability statement

The original contributions presented in the study are included in the article/supplementary material, further inquiries can be directed to the corresponding author.

## Ethics statement

The studies involving human participants were reviewed and approved by the Medical Ethics Committee of the Dalian Municipal Women and Children’s Medical Center (Group). Written informed consent to participate in this study was provided by the participants’ legal guardian/next of kin.

## Author contributions

XL and YL designed the study, performed the experiments, and were major contributors to write the manuscript. XL, NW, CB, PW, and YL performed the experiments, analyzed the data, and wrote the manuscript. All authors contributed to the article and approved the submitted version.
